# Complete genome sequence of the phage UNICOR_HM-QL, a *Berlinviru*s infecting *Escherichia coli* and *Salmonella* with potential for biocontrol in milk for Costeño cheese production

**DOI:** 10.1128/mra.00154-26

**Published:** 2026-03-05

**Authors:** Fernando Mendoza-Corvis, Pedro Marcus Pereira Vidigal, Humberto Moreira Hungaro, Omar Perez Sierra, Ana Maria Hernández Arteaga, Maryoris E. Soto Lopez

**Affiliations:** 1 Food Engineer Department, Grupo de Investigación en Propiedades y Procesos Alimentarios (GIPPAL), University of Córdoba 27987, Montería, Córdoba, Colombia; 2 Núcleo de Análise de Biomoléculas (NuBioMol), Universidade Federal de Viçosa (UFV) 28120, Viçosa, Minas Gerais, Brazil; 3 Departamento de Ciências Farmacêuticas, Faculdade de Farmácia, Universidade Federal de Juiz de Fora (UFJF) 28113, Juiz de Fora, Minas Gerais, Brazil; Queens College Department of Biology, Queens, New York, USA

**Keywords:** *Escherichia coli*, *Salmonella enterica*, polyvalent phage, Costeño cheese

## Abstract

We report the genome sequence of phage UNICOR_HM-QL, isolated from Costeño cheese, a Colombian artisanal fresh cheese produced with raw milk. This lytic phage infects *Escherichia coli* (ATCC 11229) and *Salmonella enterica* serovar Enteritidis (ATCC 13076). Comparative genomic analyses assigned UNICOR_HM-QL to the genus *Berlinvirus*. The genome comprises 39,561 bp with a GC content of 48.9% and encodes 47 coding DNA sequences (CDSs), with no tRNA genes detected.

## ANNOUNCEMENT

Costeño cheese is a traditional Colombian artisanal dairy product produced from raw milk that can harbor diverse bacterial communities, including potential pathogens (1). Bacteriophages have been investigated as biocontrol agents to reduce pathogens and spoilage bacteria in dairy products (2–7). We report here the complete genome sequence of phage UNICOR_HM-QL, a lytic phage isolated in 2023 from Costeño cheese samples collected in Colombia (Table 1).

**TABLE 1:** List of descriptors associated with the phage UNICOR_HM-QL genome^*a*^

Item	Description
Project name	Genome sequencing of the phage UNICOR_HM-QL.
Sample name	UNICOR_HM-QL.
Taxonomy ID	2732677 [*Berlinvirus* genus]
Sequencing method	Oxford Nanopore GridION
Assembly software	Flye v2.9.6-b1802 [Finished genome]
Latitude and longitude	9.231667°N, 75.819722°W
Geographic location	Colombia, Córdoba, Santa Cruz de Lorica
Collection date	2023
Environment	Sewage from Costeño cheese production
Sample volume	1,000 mL of wastewater from costeño cheese production
Sample pre-treatment	Clarification by low-speed centrifugation (3,500 rpm–15 min) followed by filtration (0.22 µm–cellulose acetate)
Propagation host	*Escherichia coli* (ATCC 11229)
Host growth medium	Brain Heart Infusion (BHI) broth
Incubation temperature	37°C
Aeration conditions	Aerobic incubation
Purification rounds	Five successive plaque purification cycles

^*a*^ All descriptors are according to recommendations of the minimum information about the genome sequences of viruses (MIGS-VI) (https://www.gensc.org/pages/standards/checklists.html).

As detailed in Table 1, phage UNICOR_HM-QL was isolated and propagated using *Escherichia coli* (ATCC 11229) as the laboratory host strain and observed to infect *Salmonella enterica* serovar Enteritidis (ATCC 13076). Genomic DNA was extracted from purified particles using the Phage DNA Isolation Kit (Norgen Biotek, Thorold, Canada). No mechanical shearing or size selection was performed prior to library preparation. Sequencing libraries were prepared with the Native Barcoding Kit 96 V14 (SQK-NBD114.96; Oxford Nanopore Technologies, Oxford, UK), and sequencing was performed on a GridION platform with a MinION/GridION R10.4.11 flow cell. Base calling was performed using the high-accuracy model v4.3.0 of Dorado v7.6.7 in MinKNOW v24.11.8. The sequencing run generated 363,699 reads (N50, 11,914 bp) assigned to the UNICOR_HM-QL barcode.

UNICOR_HM-QL reads were processed for quality control with NanoFilt v2.8.0 (8), retaining those longer than 10,000 bp with an average quality score ≥15. High-quality reads were further filtered with Filtlong v0.2.1 (https://github.com/rrwick/Filtlong), which selected the top 10% for assembly, resulting in 928 reads with an N50 of 40,777 bp. Genome assembly was conducted using Flye v2.9.6-b1802 (9), producing an estimated 646-fold coverage. Genome polishing and direct terminal repeats (DTR) identification were performed with the Nanopore DTR phage pipeline scripts (10). Briefly, iterative long-read consensus polishing was followed by read-based detection of DTR, which was used to confirm linear genome termini and genome completeness.

Genome annotation and taxonomic assignment of UNICOR_HM-QL followed the protocol described for the *Pijolavirus ufjfpfsw6* genome (11). Prokka v1.14.6 (12) was used for gene prediction; ARNold (13), FindTerm (14), and PhagePromoter v0.1.0 (15) were applied for regulatory element prediction; and VIRIDIC v1.1 (16) with the Viral Proteomic Tree (ViPTree) v.4.0 (17) was employed to calculate similarity scores against representative genomes from the International Committee on Taxonomy of Viruses (ICTV) taxonomy release MSL40.

The UNICOR_HM-QL genome comprises 39,561 bp of linear double-stranded DNA with a GC content of 48.9% and contains DTR sequences of 96 bp. The genome encodes 47 coding DNA sequences (CDSs), with a gene density of 1.19 CDSs/kb. A total of 37 promoters were predicted, including 18 host-type and 19 phage-type promoters, proposed to regulate gene expression. No tRNA genes were detected in the UNICOR_HM-QL genome.

**Fig 1:**
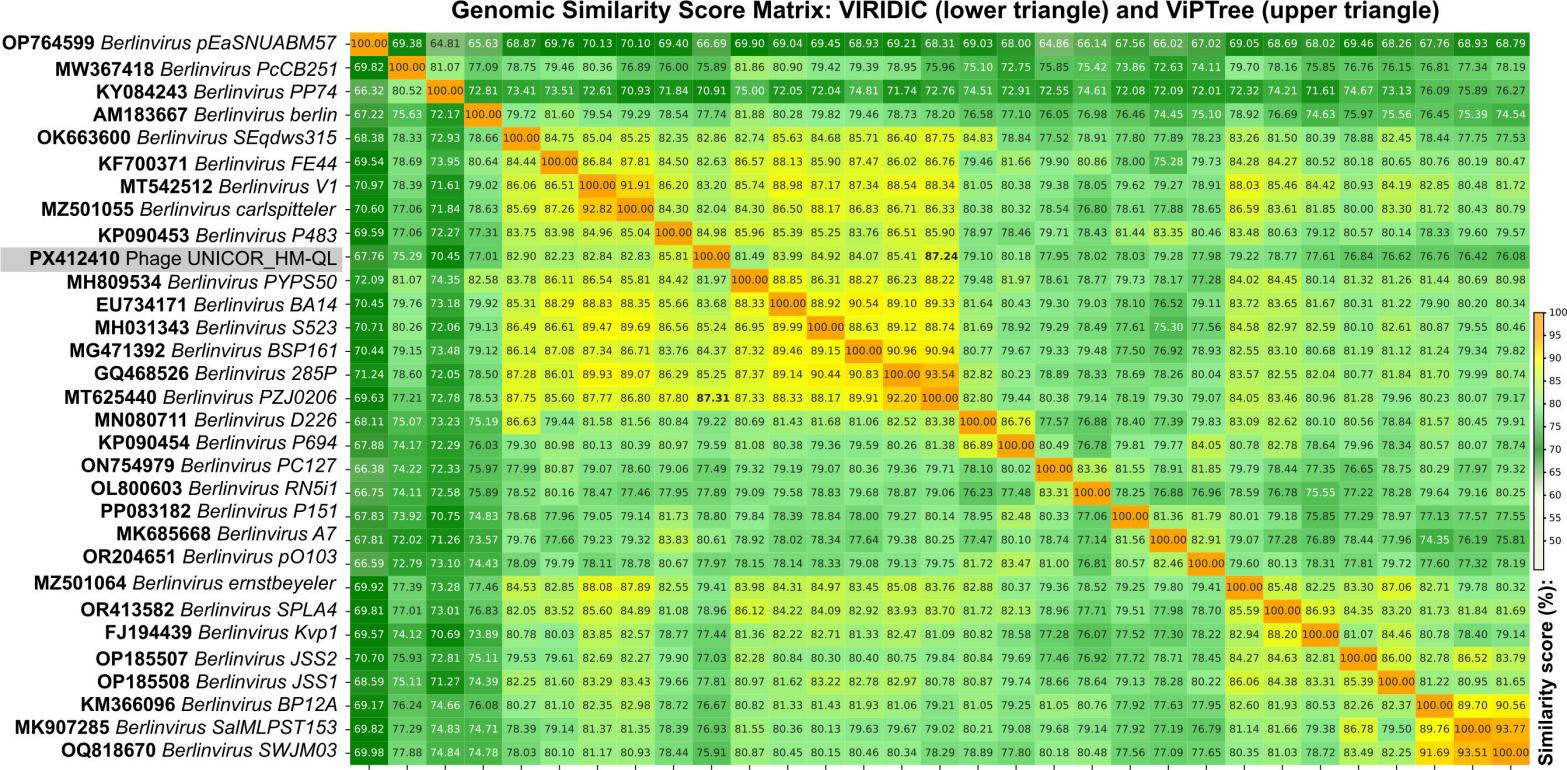
Genome distance-based taxonomy of phage UNICOR_HM-QL. The pairwise matrix summarizes the genomic similarity scores between UNICOR_HM-QL and reference species of the genus *Berlinvirus*. Similarity scores were calculated using VIRIDIC (lower triangle) and ViPTree (upper triangle) and are expressed as percentages. Values highlighted in bold indicate the highest similarity scores obtained in comparisons with UNICOR_HM-QL. A genomic similarity score of 70% represents genus demarcation threshold established by the International Committee on Taxonomy of Viruses (ICTV) Bacterial Viruses Subcommittee.

Comparative genomic analysis confirmed the high similarity of the UNICOR_HM-QL genome to phages of the *Berlinvirus*, with average similarity scores of 79.46% (VIRIDIC) and 79.42% (ViPTree), above the genus-demarcation threshold (70%) established by the ICTV (18). Among the *Berlinvirus* genomes, UNICOR_HM-QL was most similar to *Berlinvirus* PZJ0206 (GenBank accession MT625440), with scores of 87.30% (VIRIDIC) and 87.24% (ViPTree) (Fig. 1).

## Data Availability

The complete genome sequence of Berlinvirus UNICOR_HM-QL has been deposited in GenBank under accession number PX412410. The version described in this publication is the first version. The raw DNA-seq reads are available in the NCBI Sequence Read Archive (SRA) under accession number SRR36270451, associated with BioProject PRJNA1369503 and BioSample SAMN53391300.
